# New nerurological viewpoints of psychiatric sysndromes

**DOI:** 10.1192/j.eurpsy.2023.1954

**Published:** 2023-07-19

**Authors:** M. Krupa, T. Tóth

**Affiliations:** 1 Doctoral School of Education; 2 Department of Neurosurgery, University of Szeged, Szeged, Hungary

## Abstract

**Introduction:**

We research new neurological viewpoints of psychiatric syndromes in social cohesion regarding, especially the problematic of lymbic system and amygdala in adults. Nowadays, the number of the problematic use of internet is higher than before the COVID-19 pandemic. The development of the digital techniques is impulsive, which can be the main reason of most neurological dysfunctions and the change of the social communication.

**Objectives:**

In this presentation, we review studies investigating the relationship among the new digital techniques, limbic system and development of psychiatric syndromes. We attempt to provide a summary of new theories and the areas currently being researched around the topic. Another aim of our research is to present the change of the social communication and emotion regulation, which are risk factors of problematic use of internet and behavioral addictive disorders. These appear in different ways in rehabilitation and social inclusion in Europe.

**Methods:**

In order to learn about recent international results, we conducted a literature search in 4 databases ( PubMed , Medline , Web of Science, Google Scolar) using keywords (amygdala, psychiatric syndromes, adults, emotion regulation, problematic use of internet, social cohesion) over the past 5 years. From the obtained results, the English empirical journal articles were used to prepare the literature review.

**Results:**

The frequency of co-occurrence of amygdala dysfunction, problematic use of internet and behavioral addictive disorders are correlated. The studies examined the presence of symptoms of impulsivity and dopamine level of the brain primarily through cross-sectional studies. The social cohesion and inclusion regarding types are different in the regions of Europe.

**Conclusions:**

The dysfunctions of lymbic system regulation cause maladaptive emotion regulation and are risk factors and make the person vulnerable to the development of psychiatric symptoms, problematic use of internet and behavioral addictive disorders. The differents of regions and areas in Europe with the new neurological viewpoints in psychiatric disorders can help with rehabilitation in the social cohesion regarding. These changes are particularly pronounced during adolescence, when the demand for self regulation across a variety of emotional and social situations may be the greatest.

**Disclosure of Interest:**

None Declared

